# A standardized soft tissue release technique to lower the risk of greater trochanteric fractures for the anterior approach in total hip arthroplasty

**DOI:** 10.1007/s00402-021-03919-8

**Published:** 2021-05-05

**Authors:** Kilian Rueckl, Bernhard Springer, Anna Jungwirth-Weinberger, Ulrich Bechler, Maximillian F. Kasparek, Friedrich Boettner

**Affiliations:** 1 Hospital for Special Surgery, New York, NY 10021 USA; 2 Department of Orthopaedic Surgery, Koenig-Ludwig-Haus, University of Wuerzburg, Brettreichstrasse 11, 97074 Wuerzburg, Germany; 3 Department of Orthopedics, Vienna General Hospital, Medical University of Vienna, Vienna, Austria

**Keywords:** Surgical technique, DAA, Trochanteric fracture, Minimal invasive surgery, Complications

## Abstract

**Introduction:**

The direct anterior approach (DAA) is suggested to accelerate postoperative recovery and decrease the dislocation risk after primary total hip arthroplasty (THA). However, exposure of the femur can be challenging. Insufficient exposure increases the risk for intraoperative femoral fracture.

**Materials and methods:**

Of 435 consecutive anterior THA, the first 102 consecutive THA in 94 patients were treated with an external rotator tendon “release-on-demand” (RoD). The following 311 consecutive patients (333 THA) underwent routine release of the conjoint tendon (CTR) of its bony insertion on the greater trochanter only. Retrospective analysis recorded trochanteric fractures, intraoperative calcar fractures, postoperative periprosthetic fractures, stem subsidence, ossifications, and dislocations.

**Results:**

Three (2.9%) fractures of the greater trochanter were recorded in the RoD group, but no (0.0%) fractures occurred in the CTR group (*p* = 0.002). There was no significant difference in the occurrence of intraoperative calcar fractures (0% (RoD) vs. 1.2% (CTR), *p* = 0.267), postoperative periprosthetic fractures (0% (RoD) vs. 0.3% (CTR), *p* = 0.560), stem subsidence (2.0% (RoD) vs. 1.2% (CTR), *p* = 0.565) or ossifications (2.9% (RoD) vs. 1.6% (CTR), *p* = 0.344) between these groups. There were no dislocations within a minimum 12 months follow-up period.

**Conclusion:**

The routine release of the conjoined tendon (CTR group) decreases the shear forces on the tip of the greater trochanter during DAA THA and eliminates the risk of greater trochanter fractures. The routine release of the conjoined tendon did not increase the risk of postoperative dislocations.

## Introduction

Among approaches for total hip arthroplasty (THA), the direct anterior approach (DAA) has recently gained popularity. Surgeons favor its accelerated recovery and the ability for intraoperative imaging [1]. However, exposure of the femur can be more challenging compared to the posterolateral approach. Insufficient exposure increases the risk for intraoperative femoral fracture [2]. At the same time, early revision surgery is associated with the risk of deep infection of up to 33% [3]. The posterior and superior capsular release is usually necessary to mobilize the femur [4, 5]. The lateral external rotators (piriformis, gemellus superior and inferior, obturator internus and externus) pull the external rotated femur medially and posteriorly. Since in an externally rotated position the piriformis passes the trochanter more superior and the obturator externus more medial both cause much less shear force on the trochanter compared to the conjoined tendon. During external rotation and anterior mobilization of the femur, the conjoined tendon causes traction forces on the greater trochanter that can result in greater trochanter fractures. However, generous muscle release can jeopardize the benefits of the DAA [6]. This paper evaluates the following research questions: (1) Can a standardized release of the conjoined tendon (SER) reduce the risk of greater trochanter fractures in DAA THA? (2) Does a routine release of the conjoined tendon increase the dislocation rate?

## Materials and methods

### Patients

Between 9/2012 and 6/2017, the senior author performed 435 anterior THA in 405 (279 (68.9%) female and 126 (31.1%) male) patients with primary osteoarthritis (OA) or secondary OA after hip dysplasia. There were 193 left THA, 230 right THA, and 6 one-stage bilateral procedures. The mean age at time of surgery was 61.7 years (26–86, SD 9.4). The mean BMI was 25.2 kg/m2 (16.8–38.0, SD 3.6). The following femoral implants were used: 297 (68.3%) Corail ® (DepuySynthes, Warsaw IN), 72 (16.6%) Trilock, 47 (10.8%) Anthology, 13 (3.0%) Actis, 2 (0.5%) Accolade, 1 (0.2%) Synergy, 1 (0.2%) Summit, 1 (0.2%) Taperloc, 1 (0.2%) Polar stem. Acetabular components included 362 (83.2%) Pinnacle, 67 (15.4%) R3, 5 (1.1%) Trident ADM Acetabular component and 1 Trilogy (0.2%). The first 102 consecutive procedures in 94 patients were performed with a release-on-demand (RoD group) technique, where the external rotators were released only if the capsular release did not provide satisfactory access for femoral broaching. At the time the focus was on releasing the capsule itself while not releasing the external rotators. In the following 333 consecutive procedures the conjoined tendon (CTR group) was released in all patients. Demographic distributions of the two groups are displayed in Table 1. The study received IRB approval by the institutional review board at the authors institution (IRB number: 2015-071).

**Table 1:** Demographic data and statistical results for the RoD- and CTR-groups

	Treatment-group		*p* value
	RoD	CTR	
Cases	102	333	
Patients	94	311	
Female	76 (74.5%)	223 (67.0%)	
Male	26 (25.5%)	110 (33.0%)	*p* = 0.151
BMI [kg/m^2^]	25.0 (16.8–38.0)	25.3 (17.2–34.0)	*p* = 0.586
Age at date-of-surgery [years]	63.1 (43–86)	61.3 (26–86)	*p* = 0.087
Greater trochanteric fractures	3 (2.9%)	0 (0.0%)	*p* = 0.002**
Intraoperative calcar fractures	0 (0.0%)	4 (1.2%)	*p* = 0.267
Postoperative periprothetic fractures	0 (0.0%)	1 (0.3%)	*p* = 0.580
Dislocations	0 (0.0%)	0 (0.0%)	n/a
Ossifications	3 (2.9%)	5 (1.5%)	*p* = 0.344
Stem subsidence	2 (2.0%)	4 (1.2%)	*p* = 0.565

**Highly significant

### Surgical technique

All patients underwent spinal or spinal epidural anesthesia. A T-shaped capsular incision was performed in the standard fashion. #2 Etibond tagging sutures were placed in the medial and lateral capsular leaf to facilitate retraction of the capsule and later repair. After a napkin ring double osteotomy of the femoral neck and removal of the napkin ring with the leg placed in external rotation the acetabulum is reamed in a standard fashion under direct vision and the final cup is impacted using C-arm fluoroscopic guidance [1].

**Fig. 1:**
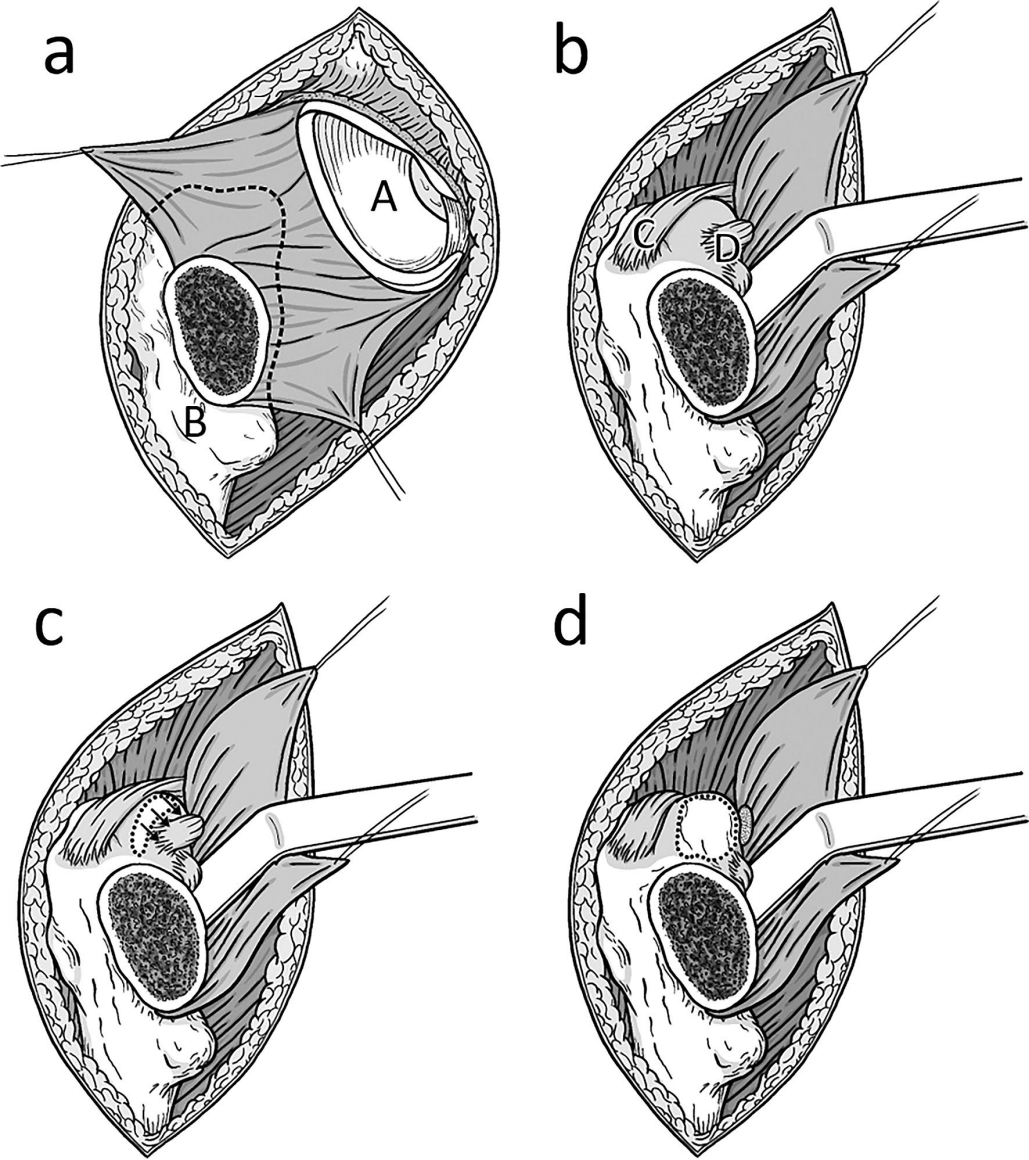
Schematic illustration of the greater trochanter: **a** after *t*-shaped incision the medial and lateral leaf of the capsule is tagged (A: acetabulum, B: femur) **b**, the lateral capsule is released from the overlying gluteus minimus and retracted medially to give access to the inside of the greater trochanter (C: piriformis muscle, D: conjoint tendon). **c** The conjoint tendon is released from the tip of the trochanter going medial. **d** After the release of the conjoint tendon the tip of the greater trochanter can be elevated without shear forces of overlaying soft tissues

**Fig. 2:**
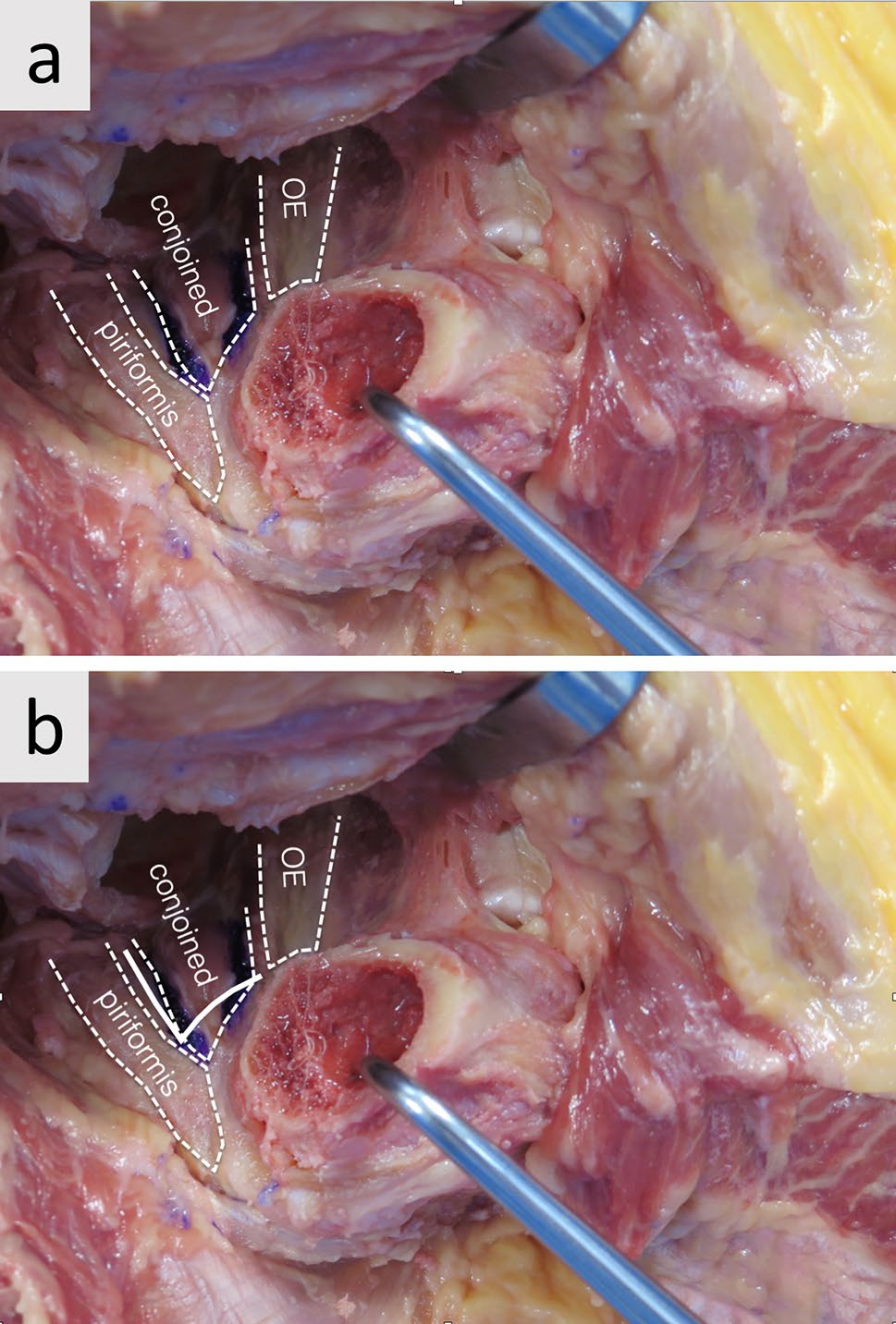
**a** and **b** show the insertion of the piriformis, conjoined tendon and obturator externus (OE) on the inside of the greater trochanter. The solid line in Fig. 2b visualizes the release which starts between piriformis and conjoined tendon over the tip of the greater trochanter and then turns medial to release the insertion of the conjoined tendon. Cleaning the tendon overlying the tip of the greater trochanter usually assures that the conjoined tendon is completely released and there is no pressure on the tip of the trochanter during elevation of the femur

The femoral release is started by elevating the gluteus minimus muscle of the lateral capsule (Fig. 1a). The capsular release is carried medial to lift the capsule off the underlying greater trochanter and expose the insertion of the external rotators (Figs. 1b, 2a). With the hook under the femur and the femur extended 30 degrees and 90 degrees of external rotation as well as 20 degrees of adduction now the conjoined tendon is released off its bony insertion using an electrocautery. The release is started separating the piriformis tendon from the conjoined tendon in line with the tip of the greater trochanter (Figs. 1c, 2b) [7]. The conjoined tendon is then released carefully avoiding the insertion of the obturator externus muscle further distal (Figs. 1d, 2b). This release was performed in all patients in the CTR group. In the release-on-demand group the external rotators were usually preserved and the release was focused on releasing the posterior and medial capsule. Finally, the leg is brought into maximum extension and the femur is elevated using the electronic hook elevator. After preparing the femoral implant, final position is confirmed under fluoroscopy and the anterior capsule is closed using #0 Vicryl.

### Postoperative management

All patients were mobilized weight bearing as tolerated. Patients did not have official hip precautions, however, patients were advised to avoid hyperextension in combination with external rotation of the leg.

### Clinical evaluation

All operative notes were analyzed for the occurrence of intraoperative trochanteric or diaphyseal femoral fractures. All postoperative radiographs were reviewed for fractures, postoperative ossifications, or stem subsidence. Diaphyseal fractures were classified based on Vancouver-classification (A1–C3), postoperative ossifications were classified based on Brooker-classification (1–4) and stem subsidence was measured in [mm] on calibrated digital radiographs using Sectra PACS software package IDS7 (Sectra AB, Linkoeping, Sweden).

The events “postoperative periprosthetic fracture”, “stem subsidence”, “ossification”, and “dislocation” were recorded with a minimum follow-up period of 1 year applicable on 102 RoD and 333 CTR cases. Data were available for all (100%) consecutive patients (mean FU 20.3 months, 12–70 months).

### Statistical analysis

Descriptive statistics were performed to describe means, range, and standard deviations for all variables. Kolmogorov–Smirnov was used to identify normal distribution of variables. Levene test was used to test for homogeneity of variances. Wilcoxon rank-sum test was performed to identify the significance for a 95% confidence interval in independent non-parametric variables. Student’s t test was performed to test independent, normal distributed, and parametric variables. Results with *p* values < 0.05 were considered as statistically significant results with *p* < 0.01 were considered as highly significant. Power calculation for an alpha failure of α = 0.05, an effect size of d = 0.4 (Difference in mean 0.02, standard deviation 0.08 ratio (group a)/ (group b) = 1/3) and an aimed power (1-β) of 95% required a sample size of group a = 73, group b = 219 patients. All statistical analyzes were performed using IBM SPSS® Statistics software version 25.0.0.0 (SPSS Inc., Chicago, Il, USA).

## Results

Three (2.9%) fractures of the greater trochanter were recorded in 102 RoD-procedures, but no (0.0%) fracture occurred in 333 CTR-procedures (*p* = 0.002). We recorded four (1.2%) intraoperative calcar fractures in the CTR group but none (0.0%) in the RoD group (*p* = 0.267). After a minimum follow-up of 1 year, there was also no significant difference in the occurrence of stem subsidence (2.0% (RoD) to 1.2% (CTR), *p* = 0.565), postoperative periprosthetic fractures (0.0% (RoD) to 1.2% (CTR), *p* = 0.580), ossifications (2.9% (RoD) to 1.5% (CTR), *p* = 0.344) or dislocations (0.0% (RoD) to 0.0% (CTR)) between these groups (Table 1).

**Fig. 3:**
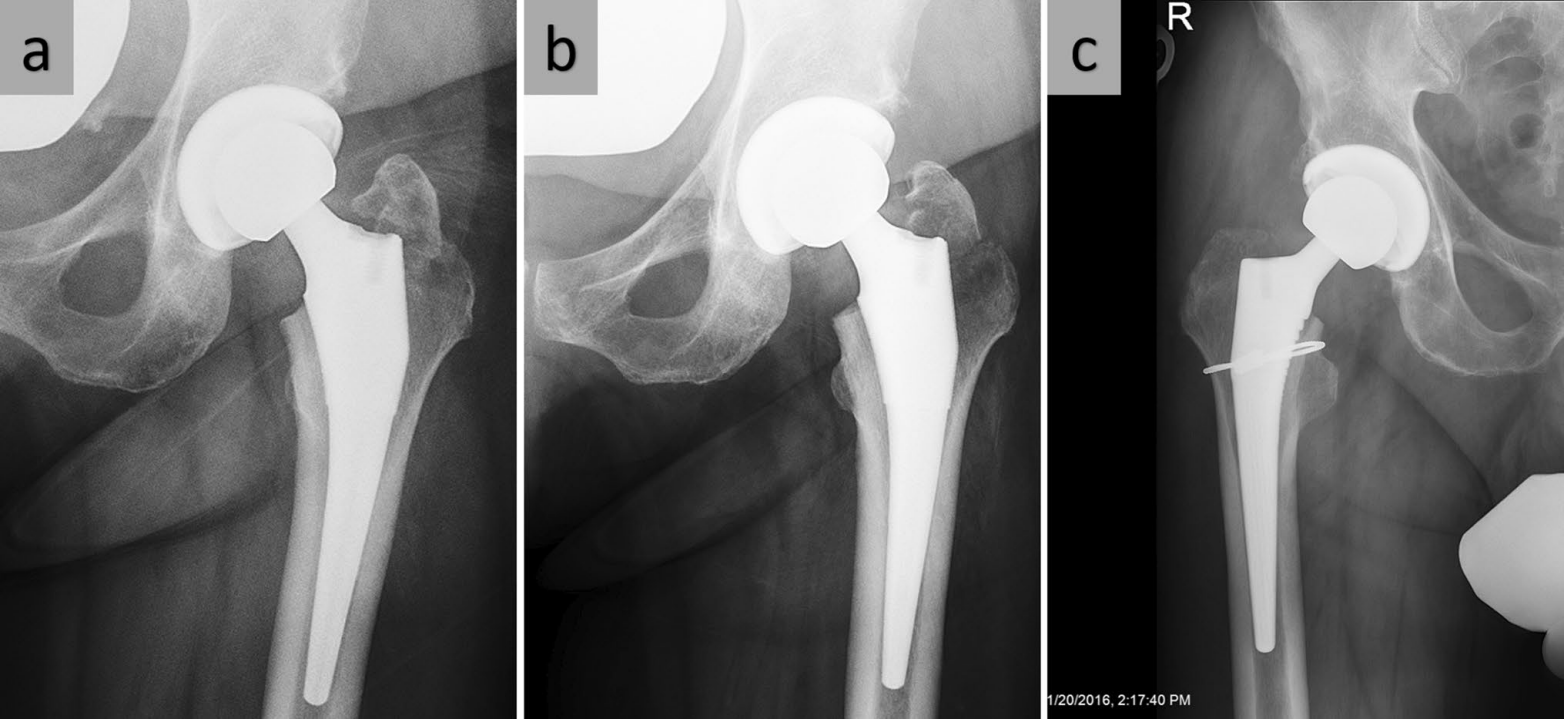
Fracture of the greater trochanter postoperatively and 4 months after surgery. **c **Intraoperative fracture with press-fit implantation of a non-cemented stem combined with a cable cerclage

All three patients with fractures of the greater trochanter showed only minor displacement of the fragment and did not require open reduction and internal fixation (Fig. 3a, b).

Threee patients with a calcar fracture underwent Dall-Miles® cables insertion around the calcar allowing for press-fit fixation of a standard uncemented femoral component. After a partial-weight-bearing period, none of the patients had any clinical symptoms or radiographic evidence of subsidence of the implant. One patient had a stable implant and additional cable fixation was not performed. In one patient, a 77-year-old female (BMI 24.3 kg/m^2^, ASA 3) who fell 2 weeks after surgery, radiographs revealed a loose stem due to a Vancouver B2 periprosthetic fracture. She was revised 13 days after the first surgery with cables and exchange of the stem to a modular uncemented revision stem (Fig. 3c).

We recorded two cases of early loosening of stems that were not associated with fractures. A 65-year-old female, (BMI 27.0 kg/m^2^, ASA 2), with a DORR C femur and osteoporosis, showed continued pain postoperatively. Postoperatively, she was mobilized with partial-weight-bearing for 8 weeks to address her pain. With persistent pain 4 months after surgery, the decision was made to revise the stem to a cemented femoral component via the posterior approach. Her postoperative recovery was uneventful, and she had no pain at the 3 months follow-up. A 58-year-old female, (BMI 18.8 kg/m^2^, ASA 2) showed a subsidence of the stem of about 2 mm. Even though there was no further migration, load-related pain persisted. CT and bone scan did not reveal clear evidence of loosening. Considering her persistent symptoms, 16 months later, revision surgery through a posterior approach was performed. Intraoperatively, the original stem seemed to be well fixed and implant loosening was not confirmed. After removal of the stem (Anthology) an anatomic uncemented stem (Synergy) was inserted and the patient’s symptoms resolved 6 months later. Subsidence of the stem with a mean of 3.5 mm (1–8 mm) was observed in another six cases. None of the patients was symptomatic or required further surgeries. Ossifications were recorded in eight cases. Three were classified as Brooker 1 and 5 as Brooker 2.

## Discussion

Inadequate exposure of the femur during DAA THA causes traction forces on the greater trochanter that can result in greater trochanter fractures. The routine release of the conjoined tendon (CTR group) decreases its shear forces on the tip of the greater trochanter during femoral elevation of a DAA THA and eliminates the risk of greater trochanter fractures in the current study. The routine release of the conjoined tendon did not increase the risk of postoperative dislocations.

Intraoperative fractures are one major reason for early revision surgery after THA. Reported complication rates up to 60% for these early revisions underline the importance to avoid such revisions under any circumstances [3]. Reducing shear forces on the greater trochanter and broaching forces on the medial calcar are important to avoid fractures. While the release of the superior and posterior capsule of the greater trochanteric has been described in the literature [4, 5] (Fig. 1a, b), the rule of the external rotator release is controversial. Anatomic studies reported that the superior capsular release has a considerable impact on the elevation of the femur [8]. The conjoint tendon insertion (common insertion of the obturator internus muscle, the gemellus superior and inferior muscle, Fig. 2a) on the inside of the femur varies but the tendon runs on the inside of the tip of the greater trochanter and elevating the femur without its release can result in considerable shear forces on the trochanter [7]. During external rotation of the femur, the tip of the greater trochanter functions as a pivot for the conjoint tendon. Its shear forces can result in intraoperative fractures of the greater trochanter (Fig. 3). In the “release-on-demand” (RoD)-group we recorded three fractures that are most likely related to over-tensioning of the conjoined tendon during elevation of the femur [5, 9]. Therefore, it is most likely that the reason for the reported fractures was an insufficient release. This argument is further supported by the fact that there were no fractures of the greater trochanter at all in the routine release of the conjoined tendon (CTR) group. It is the authors experience that releasing the conjoined tendon also greatly facilitates elevation of the femur suggesting that it is the main structure holding the proximal femur down during anterior THA. The current paper suggests that the routine conjoint tendon release helps to avoid greater trochanter fractures. Since the piriformis usually transits lateral and superior to the tip of the greater trochanter it neither causes shear forces on the greater trochanter nor does it significantly restrict the elevation of the femur (Fig. 1d). The same is true to the obturator externus that inserts more distal (Fig. 2). Both are important dynamic stabilizers of the hip [10, 11]. Some authors favor a short external rotator sparing approach to optimize stability and decrease the risk of postoperative dislocations [12]. In the current study the routine release of the conjoined tendon had no impact on the postoperative risk of dislocation. The zero percent trochanter fracture rate is lower than has been reported in the literature (1.0% of 300 patients [13], 1.0% of 1152 patients [14]).

It is important to underline these findings only apply to the DAA. In posterior approach THA, most trochanter fracture are caused by valgus forces of the broach and implant or lateral forces during removal of an implant. In DAA THA fractures of the greater trochanter are not caused by the implant itself. The fact that the conjoined tendon (obturator internus and gemelli) blocks the trochanter from elevation is illustrated in Fig. 2. All three implant types used in this study are “Banana shaped” implants with banana shaped broaches that avoid pressure on the greater trochanter. Due to difficulties moving the femur up downward pressure on the trochanter that could cause a fracture of the greater trochanter is rare. It is much more likely to enter the canal in varus threatening a calcar fracture.

There were no significant differences in the occurrence of diaphyseal (calcar) femoral fractures between the two groups. While some authors suggest that the fracture rate is lower in the posterior approach versus direct anterior approach (0% versus 2.6%; *p* = 0.04) [2] a recent meta-analysis did not reveal any differences [15]. In the authors experience calcar fractures usually occur in patients with small femur sizes and mismatch between proximal femoral anatomy and stem shape. As a result, the senior author currently uses multiple stem designs to better match the patient’s anatomy.

The current study has the following limitations: (1) This is a retrospective study. The two treatment groups were not matched. However, there was no significant difference in gender, age at time of surgery, or BMI (Table1). (2) The total number of observed complications are low. Larger study groups strengthen the statistical power. However, the current study was designed to detect differences of 2% with a power of 95% (3) All 435 DAA THA were performed by one high volume fellowship trained surgeon who does more than 250 THA per year at a specialized orthopedic hospital. (4) The study includes the learning curve of the senior surgeon and one might argue that fractures are the result of lack of experience in the early case group. However, the routine release of the conjoined tendon was developed as a result of increased early trochanter fractures rates and remains the standard surgical release techniques for the femoral release in the senior authors practice until today. (5) The paper reports intraoperative complications as well as short-term results with a minimum follow-up of 1 year. Results for a long-term follow-up are pending.

## Conclusion

The routine release of the conjoined tendon off the greater trochanter eliminates the risk of greater trochanter fractures in direct anterior THA and has no negative impact on postoperative dislocation rates.
